# A Rare Occurrence of Delayed Olanzapine-Induced Oculogyric Crisis in a Postpartum Patient: A Case Report

**DOI:** 10.7759/cureus.70127

**Published:** 2024-09-24

**Authors:** Syed Ali Bokhari, Mariam Alhosani, Louai Jalal, Alma Al Mansour, Nahid M.Elhassan

**Affiliations:** 1 Psychiatry, Al Amal Psychiatric Hospital, Emirates Health Services, Dubai, ARE; 2 Emergency Medicine, Al Qassimi Hospital, Emirates Health Services, Sharjah, ARE

**Keywords:** dose reduction, dystonia, extrapyramidal side effects, extrapyramidal symptoms, oculogyric crisis, olanzapine, postpartum psychosis, psychiatry, second-generation antipsychotics

## Abstract

Oculogyric crisis (OGC) is an acute dystonic reaction characterized by involuntary upward deviation of the eyes, often linked to the use of antipsychotic medications. While commonly associated with first-generation antipsychotics (FGAs) due to their higher propensity to cause extrapyramidal symptoms (EPS), OGC remains a rare but documented occurrence with second-generation antipsychotics (SGAs). SGAs, including olanzapine, are generally preferred in clinical practice due to their reduced risk of EPS; however, they are not completely devoid of such adverse effects.

The emergence of OGC in the context of SGAs, particularly in unique clinical scenarios, highlights the importance of awareness and careful management of potential adverse effects in clinical practice. This case report presents a rare instance of OGC in a 25-year-old postpartum woman following an independent reduction in her olanzapine dosage, a medication usually associated with a low risk of dystonia. The patient, with no previous psychiatric history, had been treated with olanzapine for postpartum depression with psychotic features and demonstrated stability.

The onset of OGC, occurring three months after the initiation of olanzapine and precipitated by stressors such as interpersonal conflicts and high-pressure situations, underscores the critical need for comprehensive patient education on the dangers of unsupervised medication adjustments. It also highlights the importance of vigilant monitoring, particularly in vulnerable populations, such as postpartum patients. This case underscores the necessity for individualized treatment approaches and highlights the rarity and clinical significance of OGC in patients on SGAs, contributing to the understanding of atypical presentations associated with these medications.

## Introduction

Oculogyric crisis (OGC) is an acute dystonic reaction characterized by involuntary upward deviation of the eyes. It is most commonly associated with the use of first-generation antipsychotics (FGAs), which are known for their higher propensity to cause extrapyramidal symptoms (EPS) [1]. However, second-generation antipsychotics (SGAs), such as olanzapine, are generally preferred in clinical practice due to their lower risk of EPS, although rare cases of OGC have been reported with these medications, which can occur unpredictably and present significant management challenges [2].

The pathophysiology of OGC involves disruption of dopaminergic pathways, particularly affecting the balance between dopamine and acetylcholine. Antipsychotic treatment can exacerbate this imbalance, leading to dystonic reactions [3]. While SGAs have a safer side effect profile compared to FGAs, factors such as abrupt dosage changes and patient-specific vulnerabilities can increase the risk of adverse reactions, including OGC [4].

Educating patients about the risks of unsupervised medication adjustments is crucial, particularly for those newly exposed to antipsychotics, and requires careful supervision [5]. Psychological stressors, such as interpersonal conflicts and high-pressure environments, can further heighten the risk of OGC, highlighting the interplay between pharmacological and psychological factors [6]. This case report contributes to the literature on the rare occurrence of OGC with SGAs like olanzapine, emphasizing the importance of vigilant monitoring, individualized treatment plans, and patient education, especially for high-risk populations. It also underscores the need for clinician awareness of these atypical but severe side effects, even with medications considered to have a safer profile [3,7].

## Case presentation

The patient is a 25-year-old Middle Eastern woman who gave birth to a healthy baby boy via normal vaginal delivery. The baby weighed 3820 grams and required brief observation in the neonatal intensive care unit (NICU) due to mild respiratory distress, which resolved without complications. The patient’s postpartum period was initially unremarkable, with no psychiatric symptoms in the first three months.

Approximately four months after delivery, the patient developed acute psychiatric symptoms that prompted a visit to a general medical facility. Given the severity and rapid escalation of her condition, a psychiatry consultation was conducted. She presented with a one-week history of pervasive low mood, severe anxiety, paranoia, insomnia, and decreased appetite. She experienced frequent panic attacks and repeatedly expressed an intense and irrational fear of dying, stating several times on the day of her presentation that she believed she was going to die, despite having no suicidal intent or plan. The patient also reported auditory hallucinations for one week, initially describing them as a male voice criticising her actions, which she later rationalised as her own internal thoughts. Her paranoia extended to delusions of being watched, leading her to take excessive security measures at home, such as keeping doors locked and blinds closed.

The patient had no personal history of psychiatric illness but had a significant family history, including bipolar disorder in her sister and paternal grandmother. Her premorbid personality was described as cheerful, independent, and sociable, with the ability to maintain positive interpersonal relationships. She had been working full-time in a desk job and was responsible for the daily care of her home, herself, and her infant without assistance.

At the time of her presentation, she was not on any psychotropic medications. Her medical regimen included calcium 400 mg/day, magnesium 150 mg/day, zinc 5 mg/day, calcium carbonate 600 mg once daily, high-dose cholecalciferol (10,000 IU and 50,000 IU weekly IV injections), an emollient moisturizer applied twice daily, iron and folic acid supplements twice daily, liposomal iron 30 mg with vitamin C 70 mg daily, a multivitamin, a prenatal vitamin, sodium chloride 0.9% nasal spray four times daily, and vitamin B1-B6-B12 complex taken with meals. She reported no current or past use of alcohol, tobacco, or illicit substances, and had no other significant medical or surgical history. Routine lab investigations were ordered but they were insignificant.

Due to the high risk and complexity of her condition, the patient was transferred to a tertiary psychiatric hospital, where she was reassessed in the emergency department and subsequently admitted. During her inpatient stay, she was diagnosed with postpartum depression with psychotic features. Her initial treatment regimen included olanzapine 10 mg once daily, sertraline 50 mg once daily, and promethazine 25 mg twice daily as needed for agitation. Over the course of her hospitalisation, the patient showed gradual improvement in her symptoms. She became more engaged in her care, with notable reductions in paranoia and anxiety. Her auditory hallucinations subsided, and her mood stabilised. Regular mental state examinations indicated progressive improvements, including better sleep and appetite, and she displayed less frequent panic symptoms. At the time of discharge following a seven-day hospitalisation, the patient was clinically stable, with no further reports of delusions or hallucinations. She was deemed fit for discharge with an adjusted treatment regimen, including an increased dose of olanzapine 15 mg once daily to ensure sustained symptom control.

She had two follow-ups in the outpatient clinic over the next two months, during which she maintained stability on the medication with no reports of any side effects. During the next follow-up clinic visit, which was three months after discharge from the inpatient, the patient presented with a new onset of the oculogyric crisis, characterised by sudden, involuntary upward eye movements and a compensatory forward-bent neck posture, impairing her ability to see properly. This symptom began approximately one month before the clinic visit. Routine lab investigations along with a comprehensive ophthalmological evaluation were conducted, both of which ruled out other potential causes and returned unremarkable findings. This condition, typically not associated with second-generation antipsychotics like olanzapine, manifested abruptly a few hours after the patient independently reduced her olanzapine dose from 15 mg to 10 mg once daily without psychiatric consultation. The episodes occurred up to five times daily, lasting up to an hour, and were temporarily alleviated by sleep but recurred with stress. Common stressors that triggered the oculogyric crisis included anxiety in situations involving interpersonal conflicts, being alone, and high-pressure environments such as driving. 

During this clinic visit, the adverse event was further substantiated using objective assessments, including the Naranjo Adverse Drug Reaction Probability Scale [8], which yielded a score of 7, suggesting a probable association between olanzapine and the development of OGC. Additionally, the Extrapyramidal Symptom Rating Scale [9] was administered, where the patient scored 6 on the dystonia subscale, thereby confirming the presence of dystonia.

Aside from the oculogyric crisis, the patient was otherwise stable. Her mental state examination revealed a good mood, with no evidence of delusions, hallucinations, or other psychotic features. She was cooperative, made good eye contact, and was oriented to time, place, and person. Her speech was coherent and relevant, her affect was appropriate, and her thought processes were logical and goal-directed. There were no signs of depressive or manic symptoms, and her insight and judgement were fair.

To manage the oculogyric crisis, the patient’s olanzapine dose was further reduced to 5 mg once daily, and she was prescribed trihexyphenidyl (benzhexol) 2 mg once daily to address the extrapyramidal symptoms. A comprehensive follow-up plan was established, including frequently scheduled follow-up calls and an in-person review within a month to monitor her response to these adjustments and ensure symptom resolution. During a follow-up phone consultation two days later, the patient reported complete resolution of the oculogyric crisis, which remained resolved in subsequent follow-up phone consultations at seven and 14 days, too. Olanzapine 5 mg once daily and trihexyphenidyl (benzhexol) 2 mg once daily were continued.

## Discussion

The occurrence of OGC, a form of acute dystonia characterized by involuntary upward deviation of the eyes, is generally associated with FGAs, though it remains quite rare with SGAs. The precise incidence of OGC remains undetermined; however, one study has reported an incidence of 5.3%. When associated with antipsychotic use, the reported incidence ranges between 0.9% and 3.4% [1]. SGAs, including olanzapine, are preferred due to their lower propensity for EPS. However, even these medications are not entirely without risk, as highlighted by the unique case of a postpartum patient who developed OGC after first-time exposure to olanzapine. This report underscores the importance of vigilance for adverse effects even with medications typically considered safer.

The incidence of OGC with SGAs, such as olanzapine, remains low but notable, as it challenges the established belief in their superior safety profile regarding EPS. A study conducted in 2015 estimated the incidence rate of OGC at 1.8% among patients treated with SGAs in a first-episode psychosis program, identifying risperidone and olanzapine as common culprits. This rate, although low, emphasizes that clinicians should not entirely dismiss the possibility of such adverse reactions, particularly in vulnerable populations such as those with no prior antipsychotic exposure [3].

The pathophysiology of OGC involves a dysregulation of dopaminergic pathways, typically exacerbated by antipsychotic treatment, which disrupts dopamine-choline balance and leads to dystonic reactions [1]. While FGAs are more commonly associated with such side effects, SGAs like olanzapine, aripiprazole, and quetiapine have also been implicated, albeit less frequently [4,10]. The mechanisms are thought to involve both D2 receptor antagonism and serotoninergic modulation, which can predispose certain patients to dystonic movements [11].

In the context of olanzapine, a number of case reports have illustrated its potential to induce OGC even at low doses. Another study conducted in 2021 documented a case where low-dose olanzapine (at 5 mg) triggered acute OGC, reinforcing the need for cautious dosing and monitoring, especially in antipsychotic-naïve patients [2]. Similar findings were reported in another study that described OGC following olanzapine use at 20 mg, suggesting a potential dose-response relationship where even therapeutic doses can precipitate such crises in susceptible individuals [12].

The clinical presentation of OGC can vary, often including not just ocular symptoms but also broader psychiatric manifestations such as visual and auditory hallucinations, panic attacks, and catatonic features [4]. This overlap with psychiatric symptoms complicates diagnosis and necessitates careful clinical evaluation to differentiate drug-induced OGC from primary psychiatric conditions.

Severe or painful oculogyric crises can be managed acutely with benztropine at a dose of 2 mg administered intramuscularly or intravenously, or with diphenhydramine at a dose of 50 mg given intramuscularly or intravenously. If there is no response, these doses can be repeated after 30 minutes. Should initial treatment prove ineffective, alternative options include diazepam, 5-10 mg intravenously, or lorazepam, 1 mg administered intramuscularly or intravenously. It is crucial to address the underlying condition, which may involve discontinuing the offending drug or reducing its dosage. For patients experiencing oculogyric crises due to neuroleptic use, short-term oral therapy with benztropine, 2-6 mg daily, or trihexyphenidyl, 4-15 mg daily, for a duration of two weeks following the acute episode, may be necessary [11,13]. There has been a report of improvement in a patient using vagus nerve stimulation. Additionally, seizures should be ruled out by performing an electroencephalogram (EEG) [14]. In refractory cases, switching to antipsychotics with a lower risk of EPS, like clozapine, may be warranted, as evidenced by several case series and reports [15].

The association of OGC with psychiatric symptoms also highlights a broader impact on treatment adherence and patient outcomes. Episodes of OGC can be distressing, leading to discontinuation of necessary medications and exacerbation of underlying psychiatric disorders. Therefore, the recognition and prompt management of OGC are crucial in maintaining therapeutic regimens and optimizing patient care.

## Conclusions

This case report highlights the rare but clinically significant occurrence of OGC associated with olanzapine, a second-generation antipsychotic generally perceived as having a lower risk of side effects like dystonia. The development of OGC in a postpartum patient, new to psychotropic medication, underscores the complexities of managing antipsychotic treatment in vulnerable populations. It demonstrates that even medications considered to have a safer profile can lead to severe reactions under certain conditions, such as abrupt dosage changes or psychological stress. This supports the need for greater awareness of the risks posed by second-generation antipsychotics and stresses the importance of vigilant monitoring, patient education, and individualized treatment strategies to prevent and address such adverse effects. It also reinforces the need for careful management and follow-up, particularly in sensitive cases like the postpartum period.
